# Reactive Hyperemia and Cardiovascular Autonomic Neuropathy in Type 2 Diabetic Patients: A Systematic Review of Randomized and Nonrandomized Clinical Trials

**DOI:** 10.3390/medicina59040770

**Published:** 2023-04-16

**Authors:** Erislandis López-Galán, Arquímedes Montoya-Pedrón, Rafael Barrio-Deler, Miguel Enrique Sánchez-Hechavarría, Mario Eugenio Muñoz-Bustos, Gustavo Alejandro Muñoz-Bustos

**Affiliations:** 1 Facultad de Medicina 2, Universidad de Ciencias Médicas de Santiago de Cuba, Santiago de Cuba 90100, Cuba; erislandislopez@infomed.sld.cu; 2 Departamento de Neurofisiología Clínica, Hospital Clínico Quirúrgico Juan Brunos Zayas Alfonso, Universidad de Ciencias Médicas de Santiago de Cuba, Santiago de Cuba 90100, Cuba; arqui6606@gmail.com; 3 Hospital Infantil Norte Juan de la Cruz Martínez Maceira, Universidad de Ciencias Médicas de Santiago de Cuba, Santiago de Cuba 90100, Cuba; rdeler33@gmail.com; 4 Departamento de Ciencias Clínicas y Preclínicas, Facultad de Medicina, Universidad Católica de la Santísima Concepción, Concepción 4090541, Chile; 5 Núcleo Científico de Ciencias de la Salud, Facultad de Ciencias de la Salud, Universidad Adventista de Chile, Chillán 3780000, Chile; 6 Departamento de Kinesiología, Facultad de Medicina, Universidad de Concepción, Concepción 4030000, Chile; marmunozb@udec.cl; 7 Escuela de Kinesiología, Facultad de Salud y Ciencias Sociales, Campus El Boldal, Sede Concepción, Universidad de las Américas, Concepcion 4030000, Chile

**Keywords:** reactive hyperemia, autonomic nervous system, diabetic autonomic neuropathy, cardiovascular autonomic neuropathy, heart rate variability, type 2 diabetes mellitus

## Abstract

*Objective*: This work aimed to determine the relationship between the autonomic nervous system and reactive hyperemia (RH) in type 2 diabetes patients with and without cardiovascular autonomic neuropathy (CAN). *Methodology*: A systematic review of randomized and nonrandomized clinical studies characterizing reactive hyperemia and autonomic activity in type 2 diabetes patients with and without CAN was performed. *Results*: Five articles showed differences in RH between healthy subjects and diabetic patients with and/or without neuropathy, while one study did not show such differences between healthy subjects and diabetic patients, but patients with diabetic ulcers had lower RH index values compared to healthy controls. Another study found no significant difference in blood flow after a muscle strain that induced reactive hyperemia between normal subjects and non-smoking diabetic patients. Four studies measured reactive hyperemia using peripheral arterial tonometry (PAT); only two found a significantly lower endothelial-function-derived measure of PAT in diabetic patients than in those without CAN. Four studies measured reactive hyperemia using flow-mediated dilation (FMD), but no significant differences were reported between diabetic patients with and without CAN. Two studies measured RH using laser Doppler techniques; one of them found significant differences in the blood flow of calf skin after stretching between diabetic non-smokers and smokers. The diabetic smokers had neurogenic activity at baseline that was significantly lower than that of the normal subjects. The greatest evidence revealed that the differences in RH between diabetic patients with and without CAN may depend on both the method used to measure hyperemia and that applied for the ANS examination as well as the type of autonomic deficit present in the patients. *Conclusions*: In diabetic patients, there is a deterioration in the vasodilator response to the reactive hyperemia maneuver compared to healthy subjects, which depends in part on endothelial and autonomic dysfunction. Blood flow alterations in diabetic patients during RH are mainly mediated by sympathetic dysfunction. The greatest evidence suggests a relationship between ANS and RH; however, there are no significant differences in RH between diabetic patients with and without CAN, as measured using FMD. When the flow of the microvascular territory is measured, the differences between diabetics with and without CAN become evident. Therefore, RH measured using PAT may reflect diabetic neuropathic changes with greater sensitivity compared to FMD.

## 1. Introduction

Type 2 diabetes mellitus (T2DM) is the predominant form of diabetes, accounting for 90% of cases [1,2]; it affects 5–10% of the general population [3] and is the leading cause of disability and death [4]. The pathophysiology of diabetes is complex and involves interactions between genetic and environmental factors [2]. The primary pathophysiology of diabetes is reportedly an interaction between metabolic and vascular dysfunction [5]. Persistent metabolic abnormalities are the main antecedents of the development of the chronic complications of diabetes, which manifest as both macrovascular and microvascular disease [6].

Data from animal and clinical models indicate that chronic hyperglycemia is the main driver of all types of diabetic microvascular disease [2], causing pathological and functional changes at the level of the microvasculature that are difficult to regulate and are dependent on endothelial, myogenic, and nerve function. The main microvascular complications of diabetes are retinopathy, nephropathy, and neuropathy [7,8].

Diabetic neuropathy (DN) is one of the most common chronic complications of diabetes [3,9,10,11] and is a leading cause of disability due to foot ulceration and amputation [10]. Quality of life is much lower among patients with DN, and the mortality rate is higher than in diabetic patients without neuropathy [8,10]. Sustained hyperglycemia can cause certain pathologic changes in nerve fibers, neuron cell bodies, the neural vasculature, and supporting connective tissue, thereby resulting in nerve damage [5]. Furthermore, it is well established that disturbances of the microvasculature play a crucial role in the degeneration of motor [2], sensory, and autonomic nerves [2,9].

### 1.1. Diabetic Autonomic Neuropathy

Diabetic autonomic neuropathy (DAN) is a complicated heterogeneous disorder that affects the autonomic neurons of the peripheral nervous system [12]. Subclinical autonomic dysfunction can occur within 1–2 years of the diagnosis of diabetes mellitus [11,13,14,15], and patients remain asymptomatic until well after the onset of diabetes [9,11,13,14]. Poor glycemic control, older age, a longer duration of diabetes, female gender, and obesity are major risk factors for the development and progression of this disease [12].

It has been suggested that DAN is a multifactorial disease where several factors combine to damage the autonomic nervous system (ANS) [7,16]. In light of this, several hypotheses associated with the pathogenesis of DAN have been formulated based on various studies. The etiological factors implicated by these hypotheses include the hyperglycemic activation of the polyol pathway [3,9,17,18,19], the hexosamine pathway [17,18], and the protein kinase C pathway [17,18,19] as well as advanced glycation [3,9,17,18,19], the generation of free radicals [3,17,18,19], oxidative stress [3,11,17,18,19], neurovascular failure [3,9,11,16,17,19], autoimmune damage [3,17,19], and neurohormonal growth factor deficiency [3,7,11,19]. Regarding the role of autoimmunity, most studies have only shown a positive association in patients with T1DM [18].

Although it has been accepted that the vascular and metabolic alterations induced by diabetes are involved in the pathogenesis of neuropathy, the disease is considered a microvascular complication due to the predominance of the ischemic pathway. The temporal relationship between microvascular disease and neuropathy has been disputed, leading to the suggestion of a cycle in which microvascular disease contributes to neuropathy that in turn promotes microvascular dysfunction [20,21]. Endoneural blood vessels exhibit cellular hyperplasia and a thickening of the capillary basement membrane [22], leading to ischemia and hypoperfusion in the nerves [23]. These changes are evident in the cutaneous microcirculation [20,23]; therefore, the microvasculature function at the skin level may reflect vascular changes at the nerve level.

Most of the body’s organ systems are dually innervated by both divisions of the ANS, i.e., the sympathetic and parasympathetic systems [13]. Damage to autonomic neurons innervating the cardiovascular system causes cardiovascular autonomic neuropathy (CAN), which may be accompanied by ischemia in the coronary territory, arrhythmias, silent myocardial infarction, severe orthostatic hypotension, and sudden death syndrome [14].

### 1.2. Diagnostic Tests for CAN

The most sensitive and specific diagnostic tests available for the evaluation of CAN are cardiovascular autonomic reflex tests (CARTs), muscle sympathetic nerve activity (MSNA), baroreflex sensitivity tests, plasma catecholamine measurement, and radioimaging to assess the sympathetic innervation of the heart [14]. Several studies have recommended CARTs to measure cardiovascular autonomic function because they are specific, sensitive, and relatively simple [24,25,26,27]. The other methods are expensive and time-consuming and require well-trained personnel [24,25,28].

Although there is a consensus that CARTs are the gold standard for diagnosing CAN, some authors found this method difficult to apply; it requires the cooperation of patients for the execution of the maneuvers, which limits its broad application in clinical practice [29]. A heart rate variability (HRV) (see Table 1) abnormality is considered one of the earliest markers of symptomatic CAN [7,30,31,32,33]. The HRV parameters that have shown the highest diagnostic value and correlations with CARTs are low frequencies (LFs) and the standard deviation of RR intervals (SDNN). Therefore, these parameters serve as important markers for the clinical assessment of CAN [29].

Subjects with CAN have a significantly reduced overall HRV compared to those without the disorder, with a mainly parasympathetic impairment appearing to precede sympathetic dysfunction [3,29,34]. Diabetic patients have been reported to exhibit sympathetic hyperactivity and an increased resting heart rate, although other authors observed low sympathetic nervous system (SNS) activity [15]. Another study suggested that sympathetic dysfunction may precede parasympathetic dysfunction, as assessed by HRV [7]. In a prospective study, Sardu C et al. [35] suggested that the link between autonomic dysfunction and an increased likelihood of a recurrence of vasovagal syncopal events in type 2 diabetic patients might be the result of excess parasympathetic tone relative to the sympathetic innervation of the heart. In summary, the association between T2DM and autonomic dysfunction is not well established and is still under investigation.

Considering that CAN dysfunction occurs in the early stages of diabetes and that multifactorial interventions can delay its development [7,15,29], a simple and effective tool for the early diagnosis of CAN is needed to improve the prognosis and quality of life of people with diabetes [7,29].

### 1.3. Reactive Hyperemia

Reactive hyperemia (RH) refers to the increase in blood flow in a given vascular territory that generally occurs in response to a period of arterial occlusion [20,36,37,38,39,40,41,42] but can also occur after the passive distension of muscles (PSRH) [43]. The mechanisms underlying this response have not been fully elucidated, but prostaglandins and other metabolic and endothelial vasodilators, as well as sensory nerves, are thought to play a role. As a relatively simple, reliable, and non-invasive test, the response to RH represents the sum of endothelium-dependent and -independent functions [20,44,45,46]. This test may also affect ANS activity, thus extending its clinical applications [36,37,38,39,40,41,47,48,49].

The most common method of assessing RH is flow-mediated dilation (FMD). This technique requires skilled operators and expensive instrumentation, and acquiring reproducible results can be challenging. For this reason, results may differ between research sites, and the application to clinical practice has been limited. Alternative methods based on the same physiologic principles quantify reactive hyperemia as a change in perfusion pressure, as measured using tonometry or laser Doppler techniques. The results obtained using these techniques have been shown to be associated with those of the FMD method in healthy young people; moreover, these methods are less dependent on a skilled operator [50].

### 1.4. Reactive Hyperemia and Diabetes

Lowered RH has been associated with diabetes [4,51]; the reduced blood glucose control seen in RH [4] precedes the late complications of diabetes [20,51,52,53]. In rats, it was confirmed that diabetes causes a decrease in basal muscle blood flow, which is also seen in response to RH, even in its earliest stages. Similarly, decreased endoneural blood flow has been reported in rats with diabetes compared to healthy controls [9]. A sensory nerve blockade decreased the skin’s reactive hyperemic response [54], and reduced vasodilation in response to heat and acetylcholine iontophoresis has been observed in the presence of diabetic neuropathy [20]. The axon-reflex-mediated neurogenic vasodilatation area was significantly smaller in type 1 diabetic patients with and without DN and was smaller in both groups of diabetic patients than in healthy subjects [55]. This work aimed to determine the relationship between the autonomic nervous system and reactive hyperemia (RH) in type 2 diabetes patients with and without cardiovascular autonomic neuropathy (CAN).

**Table 1:** Studied HRV parameters.

Parameter	Description	Physiological Significance
Linear Methods		
SDNN	Standard deviation of all RR intervals in the measurement period.	Independent indicator of frequencies, allowing the calculation of total variability [56].
RMSSD	Square root of the mean value of the sum of the squared differences of all successive RR intervals.	Reflects short-term variations in RR intervals and is used to determine the influence of the parasympathetic nervous system (PNS) on the cardiovascular system. RMSSD is directly associated with short-term variability [56].
CV	Coefficient of variation; determined by dividing the standard deviation by the mean of the RR intervals.	An indicator allowing the calculation of total variability [57,58].
Frequency methods		
HF	High frequency	These metrics are located between 0.15 and 0.4 Hz. HF is clearly related to PNS activity and has an HR-relaxant effect [56].
nHFP	Normalized HF power	
LF	Low frequency	Located between 0.04 and 0.15 Hz. These metrics are the most controversial in terms of interpretation because they can be influenced by the sympathetic nervous system (SNS) and/or PNS. In any case, it seems that long-term records provide more information about SNS activity [56].
nLFP	Normalized LF power	
LHR	LF/HF ratio	From this ratio between low and high frequencies, the sympatho-vagal balance can be evaluated. Due to the controversy in the interpretation of LF in isolation, the LF/HF ratio is used to more accurately estimate SNS activity [56].
Non-linear methods (Poincaré diagram: consecutive RR intervals are transformed into a two-dimensional scatterplot)		
SD1	Standard deviation 1	Transverse diameter of the ellipse; reflects short-term variability.
SD2	Standard deviation 2	Longitudinal diameter of the ellipse; reflects long-term variability.
SSR	SD1/SD2 ratio	Reflects the activity of the autonomic nervous system (ANS) [33].

## 2. Methodology

Research question: is there a relationship between the ANS and RH in type 2 diabetic patients with and without CAN?

Eligibility criteria: randomized and nonrandomized clinical studies characterizing reactive hyperemia and ANS activity in T2DM patients with and without CAN that were published up to September 2021 in English and Spanish were included.

Exclusion criteria: studies not conducted in type 2 diabetic patients, studies that did not evaluate the ANS and RH together, books, chapters, event reports, conference reports, case reports, animal studies, and review articles were excluded.

### 2.1. Information Sources

International database including literature with specific relevance to the study area: Medline.

Multidisciplinary bibliographic database: Scopus.

Database with an open search engine: Google Scholar.

### 2.2. Search Strategy

We reviewed studies measuring reactive hyperemia and ANS activity in T2DM patients and healthy controls. Between January 2021 and September 2021, the main article databases (PubMed, Scopus, and Google Scholar) were searched using the following keywords: “Reactive hyperemia”(all fields) AND “autonomic diabetic neuropathy”(all fields) OR “autonomic neuropathy”(all fields) AND ((“patients”(MeSH terms) OR “patients”(all fields)) AND (“diabetes mellitus, type 2”(MeSH terms) OR “type 2 diabetes mellitus”(all fields) OR “diabetes mellitus type 2”(all fields))) AND “Heart rate variability”(all fields) (number of studies: 270 articles in Google Scholar, 1 in Scopus, and 708 in PubMed). All articles compatible with our inclusion criteria were included, independent of the years of publication. To be included, case-control studies had to describe our main primary outcome, which was the measurement of reactive hyperemia and ANS activity in T2DM patients and healthy controls. We imposed no limitation on the regional origin or the nature of the control group. Studies needed to be primary research. Animal studies were excluded. Two authors (López E and Sánchez ME) conducted the literature searches, collated the articles, and extracted the data. Then, López E and Sánchez ME reviewed the abstracts independently and checked if the articles could be included in our review according to the inclusion criteria. When a consensus on suitability was not reached, a third author (Barrio R) reviewed the debated articles. Then, all authors reviewed the eligible articles.

### 2.3. Article Selection Algorithm and Search Results

**Figure 1:**
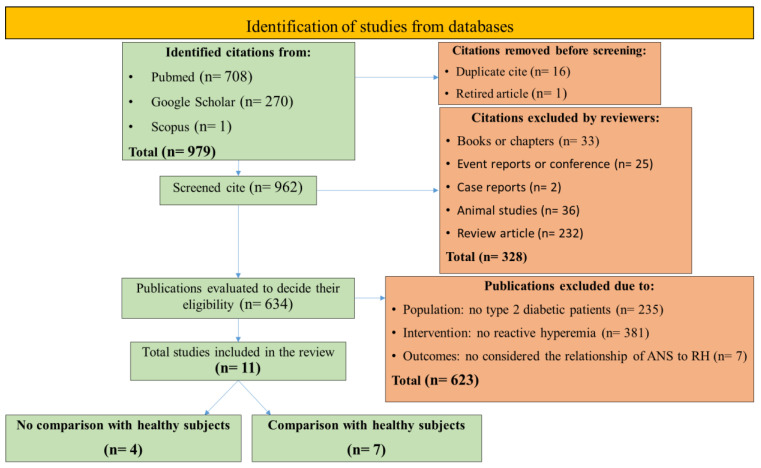
Flow diagram of the study inclusion process.

The database search revealed 979 potentially relevant citations. After removing duplicates with Zotero, 963 articles remained. One article was removed due to plagiarism. Then, 328 citations were removed because they were books or chapters (n = 33), event reports or conference reports (n = 25), case reports (n = 2), animal studies (n = 36), or review articles (n = 232). Next, 623 studies were removed because they did not meet the PICO question (population: type 2 diabetic patients, intervention: reactive hyperemia, and outcomes: ANS activity). The full texts of the 11 remaining citations were further examined and used for analysis, but only 7 had healthy control groups (comparison: healthy subjects). The search strategy is presented in Figure 1.

### 2.4. Data Analysis

To present the basic information for each study, the data were tabulated. In addition, a frequency analysis was performed using the SPSS 22.0 statistical package (SPSS Inc., Chicago, IL, USA). The tables were then analyzed to answer the research questions and identify any interesting trends.

## 3. Results

**Figure 2:**
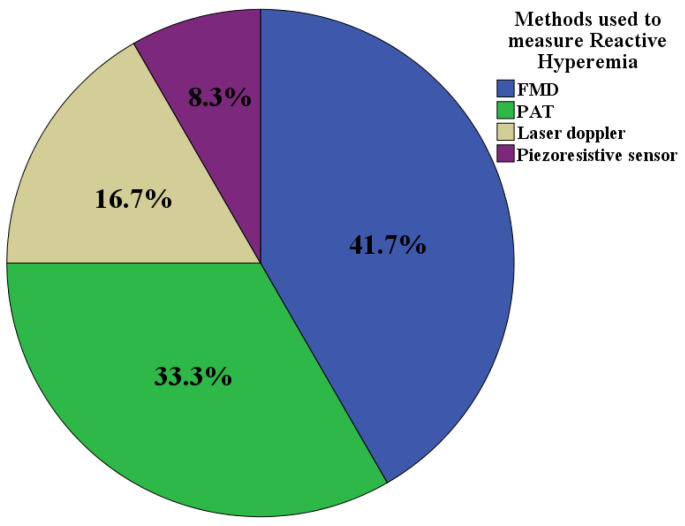
Methods used to measure reactive hyperemia (n = 11). Legend. FMD: flow-mediated dilation. PAT: peripheral arterial tonometry.

Figure 2 shows the methods used to measure RH in the analyzed studies. It can be seen that FMD was the most widely used method, followed by peripheral arterial tonometry (PAT).

**Figure 3:**
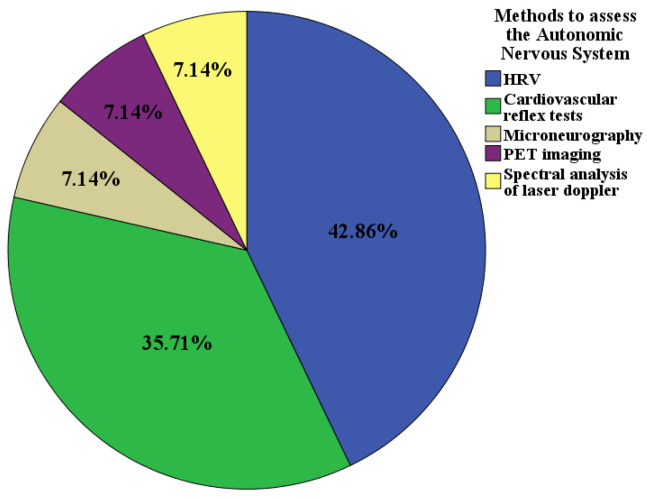
Methods used to assess the autonomic nervous system (n = 11). Legend. HRV: heart rate variability. CARTs: cardiovascular autonomic reflex tests. Microneurography of muscular sympathetic nerves. PET imaging: positron emission tomography.

Figure 3 shows the methods to assess the autonomic nervous system that were used in the analyzed studies. It can be seen that HRV was the most used method, followed by cardiovascular autonomic reflex tests (CARTs).

**Figure 4:**
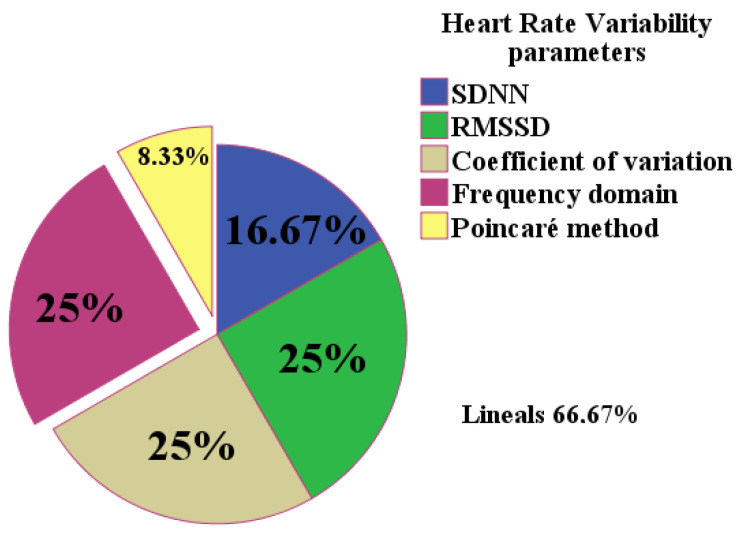
Heart rate variability evaluation parameters (n = 6). Legend. SDNN: standard deviation of all RR intervals in the measured period. RMSSD: root mean square of the sum of the squared differences of all successive RR intervals.

Figure 4 shows the evaluation parameters for HRV used in the analyzed studies. Linear methods (SDNN, the root mean square of the sum of the squared differences of all successive RR intervals, and the coefficient of variation) were the most widely used, followed by frequency methods.

**Figure 5:**
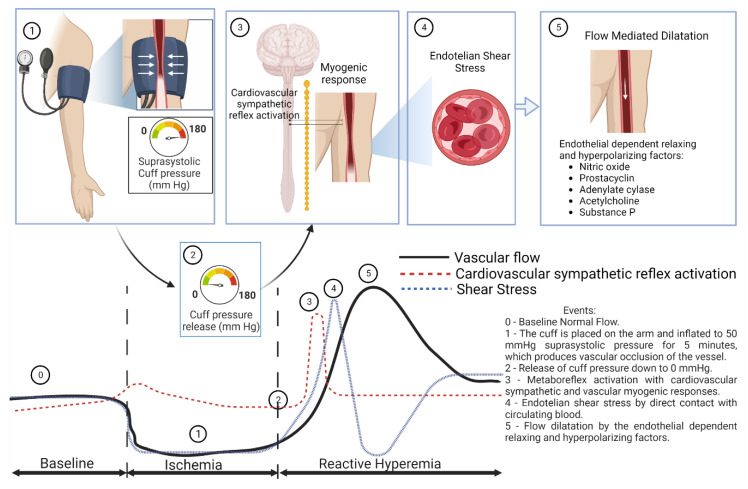
Schematic diagram about reactive hyperemia.

Figure 5 shows a schematic diagram about reactive hyperemia and its possible mechanisms.

FMD was the most widely used method to measure RH in the reviewed studies, followed by tonometry and laser Doppler techniques (Figure 2), while HRV was typically used to evaluate the ANS, followed by the CARTs (Figure 3). Regarding the latter, linear methods such as the standard deviation of RR intervals (SDNN), the root mean square of the RR intervals (RMSSD), and the coefficient of variation (CV) were the most widely used, followed by frequency methods. Only one study used non-linear methods, specifically the Poincaré diagram (Figure 4). Moreover, no consensus methodology for reactive hyperemia emerged in terms of pressure and occlusion time. An increasing number of studies were carried out on this topic between 1993 and 2020, which indicates the perceived importance of studying endothelial function and the ANS in patients with T2DM.

Of the articles reviewed herein, five showed differences in RH between healthy subjects and diabetic patients with and/or without neuropathy [15,30,33,57,58], while one study did not show such differences between healthy subjects and diabetic patients, but patients with diabetic ulcers had lower RH index values compared to healthy controls [59]. Another study found no significant difference in blood flow after a muscle strain that induced reactive hyperemia (PSRH) between normal subjects and diabetic non-smokers [43]. This different result (Table 2) may be due to the fact that a different methodology was used to induce and measure reactive hyperemia. However, there were significant differences between diabetic smokers and diabetic non-smokers and between diabetic smokers and normal subjects [43]. Therefore, there is a negative effect (independent) of tobacco on endothelial function. It is evident that, compared to healthy subjects, diabetic patients have impaired endothelial function, which seems to be better evaluated using post-occlusive reactive hyperemia than post-stretch reactive hyperemia.

Nine studies (Table 2) compared reactive hyperemia between diabetic patients with and without CAN [15,20,30,43,58,59,60,61,62]. Four of them measured reactive hyperemia using PAT [15,59,61,62], and only two found a significantly lower endothelial-function-derived measure of PAT in diabetic patients with CAN than in those without CAN [15,59]. Four studies measured reactive hyperemia using FMD. No significant differences in FMD% were reported between diabetic patients with and without CAN [30,58,60,62]. Two studies measured reactive hyperemia using laser Doppler techniques [20,43]. One of them found significant differences in the blood flow of the calf skin after a stretch between diabetic non-smokers and smokers. The diabetic smokers had significantly lower neurogenic activity at baseline than the normal subjects [43]. The greatest evidence revealed that there were no statistically significant differences in reactive hyperemia between diabetic patients with and without CAN. Therefore, the evidence of an ANS association with reactive hyperemia may depend on both the method used to measure hyperemia (microvascular or macrovascular) and that applied for the ANS examination as well as the type of autonomic deficit present in the patients (sympathetic and/or parasympathetic).

Although the FMD and the reactive hyperemia measured using tonometry (RH-PAT) measure changes in blood flow after a period of occlusion, both have differences in terms of their principles and measurement sites as well as their pathophysiological significance. The FMD measurement principle involves a one-dimensional dilation of the arterial diameter, while that of RH-PAT involves a three-dimensional measurement of volume changes within the fingertip. FMD has been better correlated with traditional risk factors, while RH-PAT has been correlated with metabolic risk factors (diabetes and obesity) and is more affected by sympathetic autonomic activation during RH. Thus, RH-PAT may detect autonomic nerve stimulation related to vascular changes. When evaluating several vascular markers, the RH-PAT is the only one that has a statistically significant relation with the electrophysiological neuropathy grading. In addition, a correlation was found between the integrity of the autonomic nervous system and RH measured using PAT but not using FMD [62].

Diabetic patients who had higher systolic blood pressure and heart rate and lower MSNA had lower endothelial function, as measured using PAT [15]. Diabetic patients with diabetic foot syndrome showed increased parasympathetic activity, decreased sympathetic activity, and decreased RH [59]. Smoking diabetic patients had a higher heart rate, less neurogenic activity, and less blood flow than non-smoking diabetics and healthy subjects [43]. Therefore, it can be argued that in the alterations in blood flow in diabetic patients during reactive hyperemia, sympathetic dysfunction plays an essential role. Reinforcing this last idea, Tuttolomondo A et al. [59] found a statistically significant negative correlation between the RH value and the parasympathetic function indices (RMSDD and HF%) and a statistically significant positive correlation with the LF/HF ratio as an expression of the sympatho-vagal balance in subjects with diabetic foot syndrome. Endothelial dysfunction and the subsequent microangiopathy of the vasa nervorum lead to decreased activity of small autonomic–sympathetic fibers. Therefore, the persistent reduction in sympathetic activity in patients with diabetic foot syndrome may be offset at the expense of increased parasympathetic activity to restore the sympatho-vagal balance.

In the analysis of the other studies that measured microvascular flow, no significant differences in RH were found between diabetic subjects with and without CAN. It must be taken into account that one study was carried out in patients with coronary artery disease [61], while the other did not distinguish between sympathetic and parasympathetic dysfunction [20].

According to Wu HT et al. [33], there were no differences in the normalized power of the LF (lnLFP), the HF (nHFP), and the low frequency–high frequency (LHR) ratio between healthy and diabetic subjects before and after RH. On the other hand, they demonstrated significant differences in the SD1/SD2 ratio (SSR) before and after RH in patients with diabetes but not in subjects without diabetes. The values of SSR 1–10 and the dilatation index were slightly correlated after RH induction, indicating that ANS impairment is associated with endothelial dysfunction. However, for this analysis, all study subjects were taken into account without distinguishing between diabetic patients and healthy subjects or the type of autonomic dysfunction. Brachial artery occlusion in healthy young women was performed to observe changes in autonomic function before and during occlusion, but no changes were noted in the HRV parameters that were measured [63]. Therefore, the impact of a post-arterial occlusion inducing changes in cardiac autonomic control remains unknown.

Seyhood G et al. [57] found that the FMD% in diabetic patients was significantly correlated with all autonomic function test values and significantly and negatively correlated with the plasma nitrite concentration. This result may indicate that the ANS and endothelial dysfunction affect each other via oxidative stress. The autonomic function test that was more correlated with the FMD% was the blood pressure response to standing, which evaluated the sympathetic system. Murata M et al. [60] reported that in diabetic patients, FMD was significantly reduced among those with erectile dysfunction compared to those without erectile dysfunction (ED). In contrast, in diabetic patients with autonomic neuropathy, there were no differences in FMD between patients with and without ED. However, there was a tendency for subjects with neuropathy to present less endothelial function, which seemed to be dependent on the type of autonomic dysfunction.

Diabetic patients in the more advanced stages of the disease (T2DM+) along the glucose continuum, based on an oral glucose tolerance test, glycosylated hemoglobin A1C, or a known history of type 2 diabetes, had higher systolic blood pressures and heart rates than individuals in the early phases. However, the baroreflex effectiveness index and the sympathetic activity were shown to be lower in the T2DM+ group. The individuals in the early phase of type 2 diabetes were shown to have increased sympathetic muscle drive, characterized by higher levels of MSNA, and subjects with less sympathetic activity had less endothelial function and a tendency to have lower RH index values [15]. Dos Santos AP et al. [30] reported less reactive hyperemia in diabetic patients with and without CAN compared to a group of healthy subjects. Meanwhile, the heart rate (HR) at rest was higher in diabetic patients with CAN compared to the other groups. Changes in the peripheral vasoconstrictor response to inspiratory metabolic reflex activation were inversely related to reactive hyperemia. The authors posited that in healthy subjects, the activation of the metabolic reflex further increases sympathetic outflow during intense exercise, thereby attenuating reactive hyperemia. In T2DM, it is possible that sympathetically mediated increases in vasoconstriction lead to a situation in which the already dysfunctional endothelium does not allow for an increase in vasodilation.

Barwick AL. et al. [20] found that the presence of neuropathy was associated with a longer latency to peak after occlusion but not with changes in the peak magnitude. HRV parameters did not predict responses. However, in other studies, flow-mediated brachial artery dilation [58] and total reactive hyperemia after passive ischemia [60] were not different between diabetic patients with and without neuropathy. Therefore, hyperemic flows were not limited by differences in cardiac sympathetic innervation. These findings differ from those of another study that reported lower hyperemic flows among diabetic patients with and without neuropathy. One possible reason for this difference, according to the authors, is that in this study patients with autonomic neuropathy had more diffuse coronary atherosclerosis than those without autonomic neuropathy [58]. However, these differences could also be due to the type and severity of autonomic dysfunction in diabetic patients. The patients in more advanced stages may present a mixed autonomic dysfunction with sympatho-vagal imbalance that makes it difficult to evaluate endothelial function using reactive hyperemia.

Low BH et al. [43] revealed that reactive hyperemia also occurs in the cutaneous microcirculation after muscle strains in healthy and diabetic subjects, but the rate of PSRH was lower in diabetic smokers compared to non-smokers. When they compared the frequency range variations related to neurogenic activity over three post-distension periods (0 s, 10 s, and 20 s), a response was induced by the distension stimulus in diabetic non-smokers but not in the healthy subjects. A possible explanation for this is that diabetic patients actually start with parasympathetic impairment and sympathetic hyperactivity, which creates an imbalance between these systems, which becomes evident after PSRH, while in healthy subjects the proper balance of the ANS avoids an exaggerated sympathetic response that can be detected using this method.

### Outlook and Limitations

Increased blood flow through arteriovenous communications bypassing capillaries (due to sympathetic denervation) was observed in diabetic patients. Arteriovenous communications that are completely innervated by the SNS are abundant within the microcirculation of the skin [7]. It has been suggested that the microcirculation of the skin should respond to any autonomic dysfunction occurring within the body [7,52,64]. Therefore, an assessment of endothelial function using tonometry can help identify patients at risk of developing diabetes [4] as well as assess cardiovascular risk [15,65] and changes in the blood pressure of the skin microcirculation due to nerve damage [7,66].

Recently, a new entropy index using an ECG synchronized with photoplethysmography (PPG) signals was used to assess the complex relationship between baroreflex sensitivity and changes in autonomic function in healthy and diabetic older subjects. The authors concluded that the decrease in entropy may provide information that will help identify T2DM patients at higher risk of developing neuropathy in the future [8,10,67]. Another study used PPG and a pulse wave variability analysis to assess ANS and endothelial function [11]. However, these papers did not assess dynamic changes in ANS and endothelial function during the RH maneuver. Moreover, the ability to quantify the extent of sympathetic damage to the cutaneous circulation through the PPG-based reactive hyperemia test (in conjunction with HRV methods) has not been fully elucidated. Finally, it is hypothesized that studying the dynamics of ANS activation during RH may provide valuable information for new treatment methods to prevent or improve the prognosis of CAN.

## 4. Conclusions

In diabetic patients, there is a deterioration in the vasodilator response to the reactive hyperemia maneuver compared to healthy subjects, which depends in part on endothelial and autonomic dysfunction. Blood flow alterations in diabetic patients during reactive hyperemia are mainly mediated by sympathetic dysfunction. The greatest evidence suggests a relationship between the ANS and RH; however, there are no significant differences in RH between diabetic patients with and without CAN, as measured using FMD. On the other hand, when the flow of the microvascular territory is measured, the differences between diabetics with and without CAN become evident. Therefore, RH-PAT may reflect diabetic neuropathic changes with greater sensitivity compared to FMD. Evidence of ANS activation after reactive hyperemia may depend both on the method used to measure hyperemia and the method applied for the ANS examination as well as on the type of autonomic deficit present in the patients.

## Figures and Tables

**Table 2:** Summary of studies that considered the relationship between the ANS and RH.

Study	Aim	RH Method	ANS Method	Results
**Studies That Made Comparisons between Healthy Subjects and Diabetic Patients**				
[33] Wu HT et al. (2013).	To assess the impacts of age and diabetes on autonomic function at baseline and during reactive hyperemia.	Piezoresistive sensor using air pressure sensation system (APSS): occlusion pressure at 200 mmHg in the upper limbs for 4 min.	Frequency analysis and non-linear Poincaré method of HRV.	Dilatation index (DI): 1.88 ± 0.54 (healthy young subjects), 1.67 ± 0.56 (healthy upper-middle-aged subjects), and 1.38 ± 0.51 (type 2 diabetic patients). There was no significant difference. Changes in SSR_(1–10)_ before and after the induction of reactive hyperemia, showing a significant difference in type 2 diabetic patients. A direct correlation between autonomic nervous system and endothelium function after RH (r = 0.29; *p* = 0.033) in all subjects of the study.
[57] Seyhood G et al. (2008).	To detect autonomic neuropathy in type 2 diabetic patients.	FMD: occlusion pressure at 250 mmHg in the upper limbs for 5 min.	Cardiovascular autonomic reflex test.	FMD% was significantly (*p* < 0.001) lower (64%) in diabetic patients in comparison to control subjects. The FMD% in diabetic patients was significantly correlated with the heart rate response to the Valsalva maneuver (Ω = 0.48; *p* < 0.01), HR variation during deep breathing (Ω = 0.59; *p* < 0.001), the immediate HR response to standing (Ω = 0.37; *p* < 0.02), the blood pressure response to standing (Ω = 0.64; *p* < 0.001), and the blood pressure response to hand gripping (Ω = 0.41; *p* < 0.01). FMD% was significantly (*p* < 0.001) and negatively (r = −0.67) correlated with the plasma nitrite concentration.
[15] Baqar S et al. (2019).	To compare sympathetic nervous system activity and endothelial function based on low sodium intake.	Peripheral arterial tonometry: suprasystolic occlusion pressure at 60 mmHg or 200 mmHg in the upper limbs for 5 min and 5–10 min post-occlusion.	Muscle sympathetic nerve activity (MSNA)	The diabetic patients with cardiometabolic risk factors (T2DM+) had higher systolic blood pressures (*p* = 0.04) and heart rates (*p* = 0.002) than normal glucose tolerance (NGT), impaired glucose tolerance (IGT) and treatment-naive diabetic patients (T2DM−). The endothelial-function-derived measure of the PAT ratio (*p* = 0.04) was lower in the T2DM+ group, and the reactive hyperemic index trended towards being lower in the T2DM+ group (*p* = 0.08). Despite the lower burst incidence, the baroreflex effectiveness index was shown to be lower in the T2DM+ group compared to all other groups (*p* = 0.0002).
[30] Dos Santos AP et al. (2015).	To evaluate the effects of inspiratory loading on blood flow in diabetic patients.	FMD: occlusion pressure at 250 mmHg in the upper limbs for 5 min.	Cardiovascular autonomic reflex test	FMD% was decreased in both T2DM (6 ± 2) and T2DM with CAN (4 ± 3) patients compared to healthy subjects (12 ± 2). Resting HR was higher (*p* < 0.05) in T2DM with CAN (80 ± 7) compared to healthy subjects (68 ± 6), and it had a trend to be higher than in T2DM.
[58] Di Carli MF et al. (1999).	To evaluate the sympathetic regulation of myocardial blood flow in diabetic patients with autonomic neuropathy.	FMD: occlusion pressure at 300 mmHg in the upper limbs for 5 min.	Cardiovascular autonomic reflex test and radioimaging of the sympathetic innervation of the heart.	FMD% was lower in diabetic patients with (7.0 ± 3.4; *p* = 0.018) and without (7.8 ± 8.1; *p* = 0.045) sympathetic nerve dysfunction than in the healthy volunteers (13.7 ± 4.1). FMD% was similar in both groups of diabetic patients.
[43] Low BH et al. (2020).	To investigate the microvascular response of the skin to muscle stretching stimuli in diabetic smokers.	Laser Doppler: reactive hyperemia in the cutaneous microcirculation after a 10 s stretching stimulus.	Spectral analysis of laser Doppler.	After a 10 s stretching stimulus, blood flow was significantly elevated within the first 10 s, followed by a gradual decrease. The blood flow of the calf skin after the stretch was not significantly different between normal subjects and diabetic non-smokers; however, there were significant differences between diabetic smokers and diabetic non-smokers (*p* < 0.01) and between diabetic smokers and normal subjects (*p* < 0.01). The intensity of the spectrum of the neurogenic activity reached its maximum value immediately after the stretching stimulus (*p* < 0.05) and then decreased gradually in the next periods for diabetic subjects, while there was almost no neurogenic response for the normal subjects. Compared to the normal subjects, the intensity of the neurogenic activity at baseline was significantly lower for smokers (*p* < 0.05).
[59] Tuttolomondo A et al. (2021).	To evaluate the alteration of the sympatho-vagal balance and correlation with endothelial dysfunction in patients with diabetes mellitus with and without diabetic foot syndrome and in healthy subjects.	Peripheral arterial tonometry: suprasystolic occlusion pressure at 60 mmHg or 200 mmHg in the upper limbs for 5 min.	HRV (SDNN, RMSSD, and spectral analysis).	Diabetic patients with diabetes foot syndrome (DFS) showed a higher HF (21.23 ± 14.68 vs. 11.10 ± 11.58; *p* = 0.002), a lower LF/HF ratio (1.63 ± 1.66 vs. 3.18 ± 2.82; *p* = 0.001), and a lower RH (1.60 ± 0.33 vs. 2.01 ± 0.69; *p* < 0.0005) than the diabetic controls and a lower RH (1.60 ± 0.33 vs. 2.20 ± 0.38; *p* = 0.00) and a similar HF% (21.23 ± 14.68 vs. 14.70 ± 11.15; *p* = 0.82) and LF/HF ratio (1.63 ± 1.66 vs. 1.07 ± 0.44; *p* = 0.59) compared to the healthy controls. Diabetic control patients showed a higher LF/HF ratio (3.18 ± 2.82 vs. 1.07 ± 0.44; *p* < 0.0005) and a comparable RH (2.01 ± 0.69 vs. 2.20 ± 0.38; *p* = 0.315) compared to the healthy controls. In DFS, there was a statistically significant negative correlation between the RH and RMSDD (Pearson index: −0.47; *p* = 0.0001), the standard deviation (Pearson index: −0.374; *p* = 0.002), and HF% (Pearson index: −0.395; *p* = 0.001) and a positive correlation with the LF/HF ratio (Pearson index: 0.280; *p* = 0.026).
**Studies That did not compare healthy subjects and diabetic patients**				
[60] Murata M et al. (2012).	To determine the relationship between endothelial dysfunction and erectile dysfunction (ED) in type 2 diabetic patients.	FMD: suprasystolic occlusion pressure at 50 mmHg in the upper limbs for 5 min.	HRV (CV of RR intervals)	FMD was significantly lower (*p* = 0.038) in the diabetic patients with ED (2.84 ± 0.34%) than in those without ED (3.82 ± 0.39%). In the diabetic patients without autonomic neuropathy, FMD was significantly reduced in the diabetic patients with ED compared to those without ED (2.43 ± 0.38% vs. 3.92 ± 0.41%, *p* = 0.015). In contrast, in the diabetic patients with autonomic neuropathy, there was no difference in FMD between the diabetic patients with and without ED.
[61] Hartmann A et al. (1993).	To investigate the pain threshold and reactive hyperemia in diabetic patients with and without autonomic neuropathy and in patients with silent myocardial ischemia.	Peripheral arterial tonometry: suprasystolic occlusion pressure in the upper limbs for 5 min.	HRV at rest and during deep breaths (CV of RR and RMSSD intervals)	The maximum hyperemia was obtained 15 s after the deflation of the cuff, and hyperemia returned to baseline 300 s after the termination of forearm ischemia. Total post-ischemic (with exercise) reactive hyperemia was significantly higher (*p* < 0.01) in patients with silent myocardial ischemia (219.56 ± 90.5 mL/l00 mL of tissue) compared to diabetic patients with neuropathy (179.6 ± 79 mL/100 mL of tissue) and without neuropathy (180.8 ± 58 mL/100 mL of tissue). There was no significant difference in total reactive hyperemia in diabetic patients with and without neuropathy.
[20] Barwick AL et al. (2016).	To examine the relationships between clinically detectable peripheral sensory neuropathy, cardiac autonomic dysfunction, and RH.	Laser Doppler: occlusion pressure at 220 mmHg in the upper limbs for 3 min and 4 min of post-occlusion recording.	HRV (SDNN, RMSSD, and spectral analysis).	The presence of neuropathy was associated with a longer latency to peak after occlusion but not with changes in the peak magnitude. HRV parameters did not predict responses.
[62] Ando A et al. (2021).	To investigate the association of RH and other physiological vascular markers with neuropathy markers.	Peripheral arterial tonometry and FMD: occlusion of the non-dominant arm maintained for 5 min above 200 mmHg or at least 50 mmHg above the systolic blood pressure.	CV of RR intervals at rest and during deep breathing.	RH had no significant statistical different between diabetic patients with (FMD: 1.6 ± 1.2; PAT: 1.7 ± 0.5) and without neuropathy (FMD: 1.8 ± 1.3; *p* = 0.39; PAT: 1.7 ± 0.4; *p* = 0.78). RH-PAT had significant statistical relations with electrophysiological grading (Spearman’s coefficients: 0.22; *p* < 0.05).

RH: reactive hyperemia, ANS: autonomic nervous system, RMSSD: root mean square of the RR intervals, SDNN: standard deviation of RR intervals, CV: coefficient of variation.

## Data Availability

Not applicable.

## Abbreviations

ANS	Autonomic nervous system
CAN	Cardiovascular autonomic neuropathy
CARTs	Cardiovascular autonomic reflex tests
CV	Coefficient of variation
DAN	Diabetic autonomic neuropathy
DM	Diabetes mellitus
DM2	Diabetes mellitus type 2
DN	Diabetic neuropathy
ECG	Electrocardiogram
ED	Erectile dysfunction
FMD	Flow-mediated dilation
HDL-c	High-density lipoprotein
HF	High frequency
HR	Heart rate
HRV	Heart rate variability
LDL-c	Low-density lipoprotein
LF	Low frequency
LHR	Low frequency–high frequency ratio
MSNA	Muscle sympathetic nerve activity
nHFP	Normalized high-frequency power
nLFP	Normalized low-frequency power
PAT	Peripheral arterial tonometry
PET	Positron emission tomography
PNS	Parasympathetic nervous system
PPG	Photoplethysmography
PSRH	Post-stretch reactive hyperemia
RH	Reactive hyperemia
RMSSD	Root mean square of the RR intervals
SBP	Systolic blood pressure
SD1	Standard deviation 1
SD2	Standard deviation 2
SDNN	Standard deviation of RR intervals
SNS	Sympathetic nervous system
SSR	SD1/SD2 ratio
TtP	Pulse transit time
